# Iodine and thyroid status during pregnancy and risk of stillbirth: A population‐based nested case–control study

**DOI:** 10.1111/mcn.13252

**Published:** 2021-08-04

**Authors:** Alexandra C. Purdue‐Smithe, Tuija Männistö, Elijah Reische, Kurunthachalam Kannan, Un‐Jung Kim, Eila Suvanto, Heljä‐Marja Surcel, Mika Gissler, James L. Mills

**Affiliations:** ^1^ Division of Intramural Population Health Research, National Institutes of Health *Eunice Kennedy Shriver* National Institute of Child Health and Human Development Bethesda Maryland USA; ^2^ Northern Finland Laboratory Center NordLab Oulu University Hospital Oulu Finland; ^3^ Department of Pediatrics and Department of Environmental Medicine New York University School of Medicine New York New York USA; ^4^ Department of Earth and Environmental Sciences University of Texas at Arlington Arlington Texas USA; ^5^ Oulu University Hospital, Department of Children and Women and Oulu University Medical Faculty PEDEGO Research Unit Medical Research Center Oulu Finland; ^6^ Biobank Borealis of Northern Finland Oulu University Hospital Oulu Finland; ^7^ Faculty of Medicine University of Oulu Oulu Finland; ^8^ Information Services Department Finnish Institute of Health and Welfare Helsinki Finland; ^9^ Department of Neurobiology Karolinska Institute Stockholm Sweden

**Keywords:** iodine, pregnancy, pregnancy loss, stillbirth, thyroglobulin, thyroid hormones, thyroid‐stimulating hormone

## Abstract

Prior research suggests that severe iodine deficiency in pregnancy may be associated with stillbirth. However, the relationship between mild to moderate iodine insufficiency, which is prevalent even in developed countries, and risk of stillbirth is unclear. We thus examined associations of iodine status and risk of stillbirth in a prospective population‐based nested case–control study in Finland, a mild to moderately iodine insufficient population. Stillbirth cases (*n* = 199) and unaffected controls (*n* = 249) were randomly selected from among all singleton births in Finland from 2012 to 2013. Serum samples were collected between 10 and 14 weeks gestation and analysed for iodide, thyroglobulin (Tg) and thyroid‐stimulating hormone (TSH). Odds ratios (ORs) and 95% confidence intervals (CIs) for stillbirth were estimated using logistic regression. After adjusting for maternal age, prepregnancy body mass index, socio‐economic status and other factors, neither high nor low serum iodide was associated with risk of stillbirth (Q1 vs. Q2–Q3 OR = 0.92, 95% CI = 0.78–1.09; Q4 vs. Q2–Q3 OR = 0.78; 95% CI = 0.45–1.33). Tg and TSH were also not associated with risk of stillbirth in adjusted models. Maternal iodine status was not associated with stillbirth risk in this mildly to moderately iodine‐deficient population. Tg and TSH, which reflect functional iodine status, were also not associated with stillbirth risk. The lack of associations observed between serum iodide, TSH and Tg and risk of stillbirth is reassuring, given that iodine deficiency in pregnancy is prevalent in developed countries.

**Key Messages**

- Severe iodine deficiency may lead to adverse pregnancy outcomes. Mild to moderate iodine deficiency during pregnancy is common in developed countries, but it is unclear whether it increases the risk for stillbirth.
- Women who experienced stillbirths did not differ significantly in iodine, Tg or TSH concentration from those who had live births in a population with mild to moderate iodine deficiency suggesting that deficiency levels seen in developed countries do not affect thyroid function or increase the risk of stillbirth.

## 1 INTRODUCTION

An estimated 2.6 million stillbirths occur worldwide each year (de Bernis et al., 2016). Even in high‐income countries, the stillbirth rate ranges from 1.3 to 8.8 per 1000 births (de Bernis et al., 2016). Established risk factors for stillbirth include placental abnormalities, congenital anomalies, infection and fetal growth restriction (Reinebrant et al., 2018); however, the cause of stillbirth is frequently unknown (de Bernis et al., 2016).

One possible risk factor for stillbirth is maternal iodine deficiency. Iodine, an essential nutrient found primarily in fish, eggs, dairy products and iodized salt (Nyström et al., 2016), is required for production of triiodothyronine (T3) and thyroxine (T4), the thyroid hormones. Synthesis of T4 and T3 is tightly regulated by the hypothalamic–pituitary–thyroid axis via thyroid‐stimulating hormone (TSH) and requires the iodination of thyroglobulin (Tg) in the follicular lumen of thyrocytes (Zimmermann et al., 2008). Iodine deficiency is especially prevalent among pregnant women, even in developed countries (Gizak et al., 2017), because the fetus is completely dependent on the maternal iodine supply (Leung et al., 2011). Haemodilution, increased renal clearance of inorganic iodide and oestrogen‐stimulated production of Tg further contribute to increased iodine requirements during pregnancy, resulting in higher risk of deficiency among pregnant women (Leung et al., 2011). Iodine insufficiency in pregnancy can lead to thyroid dysfunction, which is associated with risk of pre‐eclampsia, pregnancy loss, congenital anomalies and possibly stillbirth (Andersen et al., 2014; Casey et al., 2007; Nijkamp et al., 2016; Su et al., 2011).

Reports of reductions in stillbirth rates following prenatal iodine supplementation can be found as far back as the 1930s (Kemp, 1939; Potter et al., 1979). Some epidemiologic research suggests that women with low urinary iodine concentrations (UIC) are at greater risk of stillbirth (Dillon & Milliez, 2000), but other studies have found no association between iodine levels and stillbirth risk (Charoenratana et al., 2016; Oral et al., 2016; Yang et al., 2018). Importantly, most previous studies were conducted in developing countries, where iodine deficiency is more severe and often co‐occurs with other nutritional deficiencies, potentially confounding the effect estimates for iodine status and stillbirth. Little information is available on the relationship between stillbirth and mild to moderate iodine deficiency in pregnancy, which is common in many developed countries (Gizak et al., 2017). Moreover, prospective studies reporting no associations of iodine status and stillbirth ascertained extremely few cases (Charoenratana et al., 2016; Yang et al., 2018), given the relative rarity of stillbirth as a pregnancy outcome. Adequately powered prospective studies are needed to address this important research gap.

To address the limitations of prior work and determine whether iodine and thyroid status are associated with risk of stillbirth, we conducted a population‐based nested case–control study among all pregnant women in Finland, where iodine insufficiency is common but most other nutritional deficiencies are not (Ittermann et al., 2020; Nyström et al., 2016; Viñas et al., 2011).

## 2 METHODS

### 2.1 Study population

As described previously (Bell et al., 2019; Purdue‐Smithe et al., 2019), we conducted a population‐based nested case–control study within the Finnish Maternity Cohort, using the Finnish Medical Birth Register to ascertain pregnancy and perinatal outcome data. Beginning in 1983, the Finnish Maternity Cohort has collected more than two million serum samples from more than 950,000 pregnant women living in Finland, which reflects ~98% coverage of the pregnant population. Blood samples were collected to screen for hepatitis B, human immunodeficiency virus, syphilis and rubella antibodies. The samples were drawn in general between 10 and 14 weeks gestation at local maternity care units and sent to the prenatal serology laboratory of the Finnish Institute for Health and Welfare in Oulu. There, sera were separated by centrifugation, screening analyses were performed and the remaining serum (1–3 ml) was stored at −25°C.

Biochemical data were linked to clinical data from the Finnish Medical Birth Register via unique personal identification numbers given to all Finnish citizens and residents at birth or at time of permanent residence. The Finnish Medical Birth Register includes data on all live births and stillbirths in Finland with a birthweight ≥ 500 g or a gestational age at birth ≥ 22 gestational weeks. Maternal data collected by the Finnish Medical Birth Register include age, height and prepregnancy weight, socio‐economic status based on self‐reported occupation, marital status, pregnancy history, smoking status and other factors. Data collected on infants include sex, gestational age at birth and birth height and weight.

### 2.2 Case and control ascertainment

We randomly selected 200 cases of stillbirth and 250 potential controls from among all singleton births in the entire country between 2012 and 2013 with available serum samples in the Finnish Maternity Cohort. Because the 250 potential controls were randomly selected without regard to case/control status, one control pregnancy ended in stillbirth and was thus reclassified as a case. After reclassification, the study included 201 cases and 249 controls. Women with known thyroid disease were excluded yielding a final sample size of 199 cases and 249 controls.

### 2.3 Measurement of iodide, Tg and TSH

Details regarding serum iodide measurement in this study population have been published previously (Bell et al., 2019). Briefly, 200‐μl serum samples were thawed at room temperature, vortexed and transferred to polypropylene tubes and fortified with a known concentration of internal standard (^35^Cl^18^O_4_^−^). Samples were pretreated (by the addition of acetic acid‐ascorbic acid followed by digestion with tetramethyl ammonium hydroxide), centrifuged and analysed by high‐performance liquid chromatography (Alliance 2695 HPLC) coupled with electrospray triple‐quadrupole mass spectrometry (Micromass, ESI–MS/MS; Waters Corporation, Milford, MA, USA). Identification and quantification of iodide was performed using electrospray negative ionization multiple reaction monitoring. Using an anion exchange column, AS‐21, iodide was separated from other constituent cations and anions in the sample matrix by using methylamine mobile phase. A relative response of native compounds to the labelled internal standard was used for quantification. The limits of quantitation determined for iodide in human blood sera was 0.25 ng/ml. Serum Tg and TSH concentrations were measured using a commercial immunoassay (Siemens AG, Munich, Germany), as they have been shown to be reliable markers of thyroid function in pregnant women (Roti et al., 1991). The intra‐assay and interassay coefficients of variation for Tg were <8% and <12%, respectively, and for TSH < 5% and <5%, respectively. Because serum iodine and Tg concentrations differ based on the laboratory method and population, we constructed our own normal range using the 249 normal controls. Thus, we were able to determine whether stillbirth cases differed significantly from normal pregnant women with regard to iodine and thyroid status in the same population. The normal range for serum iodide (Quartiles 2–3) was 6.7–38.3 μg/L and for Tg (≤75th percentile) was ≤35.6 μg/L. We also considered a higher cutpoint (≤95th percentile: ≤83.6 μg/L) to define the normal reference range for Tg and lower cutpoint (≥5th percentile: 1.34 μg/L) to define the normal reference range for serum iodide.

### 2.4 Statistical analysis

Characteristics of the cases and controls were compared using *t*‐tests for continuous variables and *χ* ^2^ tests for categorical variables. For analyses evaluating continuous exposures, serum iodide, Tg and TSH values were normalized by log‐transformation. Participants were divided into quartiles of iodide and Tg based on the distribution of these biomarkers in the control group, allowing us to construct our own normal range of serum iodide and Tg specific to this population. For TSH, we categorized participants as having high TSH (>3.1 and >3.5 mIU/L in the first and second trimesters, respectively), normal TSH (0.1–3.1 and 0.2–3.5 in the first and second trimesters, respectively) or low TSH (<0.1 and <0.2 mIU/L in the first and second trimesters, respectively), according to previously defined reference ranges for pregnant Finnish women (Männistö et al., 2011).

Using logistic regression, we estimated unadjusted odds ratios (ORs) and 95% confidence intervals (CIs) for stillbirth according to serum iodide, Tg and TSH. In adjusted models, we estimated ORs and 95% CIs while controlling for maternal age, maternal body mass index, socio‐economic status, smoking status, parity and marital status. Individuals with missing data on body mass index (*n* = 7) were dropped from multivariable analyses.

Because we used samples collected during the pregnancy, we were able to identify some previously undiagnosed thyroid abnormalities by TSH assay. In sensitivity analyses, we excluded women with abnormal TSH levels during the pregnancy to determine whether iodide may be associated with stillbirth among women without overt hyperthyroidism or hypothyroidism. To determine whether our estimates were robust to confounding by other adverse pregnancy outcomes, we conducted sensitivity analyses excluding women diagnosed with pre‐eclampsia and gestational diabetes mellitus (GDM). We also evaluated potentially non‐linear associations between iodine status and stillbirth using restricted cubic spline models. All analyses were run using SAS, version 9.4 (SAS Institute Inc., Cary, NC, USA).

### 2.5 Ethical considerations

This study was conducted according to the guidelines set in the Declaration of Helsinki, and all procedures involving research study participants were approved by the steering committee of the Finnish Maternity Cohort, the ethical review boards of the Northern Ostrobothnia Hospital District and the Finnish Institute for Health and Welfare, Oulu, Finland, and the Office of Human Subjects Research, National Institutes of Health, Bethesda, MD, USA (#13459). Written informed consent was obtained from all subjects.

## 3 RESULTS

Among women in the study sample, stillbirth cases were older, more likely to be smokers and less likely to be married or cohabiting with their partners than controls (Table 1). Compared with controls, stillbirth cases also had higher gravidity and shorter gestations and were screened earlier in pregnancy.

**Table 1:** Characteristics of stillbirth cases and controls: Finnish Maternity Cohort, 2012–2013

Characteristic	Cases (*n* = 201)	Controls (*n* = 249)	*P* value^a^
Maternal age (years)	30.6 ± 6.1	29.4 ± 5.3	0.03
Prepregnancy BMI (kg/m^2^)	25.8 ± 5.8	24.8 ± 4.9	0.10
Gravidity	2.0 ± 2.4	1.4 ± 1.8	0.05
Parity	1.4 ± 1.9	1.1 ± 1.6	0.16
Nulliparous	85 (43)	108 (43)	0.85
Gestational age at screening (weeks)	11.5 ± 3.9	10.8 ± 2.9	0.05
Gestational age at birth (weeks)	33.0 ± 6.3	39.6 ± 1.5	<0.01
Thyroglobulin (μg/L)^b^	22.8 ± 29.6	23.1 ± 22.1	0.50
Serum iodide (μg/L)^b^	17.5 ± 28.3	18.8 ± 31.6	0.67
Thyroid‐stimulating hormone (mIU/L)^b^	1.2 ± 0.9	1.1 ± 1.0	0.79
Smoking status			<0.01
Non‐smoker	141 (70)	204 (82)	
Smoker	40 (19)	40 (16)	
Unknown	20 (10)	5 (2)	
Socio‐economic status			<0.01
Blue collar	20 (10)	31 (13)	
Lower white collar	32 (16)	67 (27)	
Upper white collar	9 (5)	28 (11)	
Entrepreneur	2 (1)	10 (4)	
Student	14 (7)	25 (10)	
Other/unknown	124 (62)	88 (35)	
Chronic hypertension	3 (2)	3 (1)	0.99
Gestational hypertension	4 (2)	6 (2)	0.99
Pre‐eclampsia	5 (3)	4 (2)	0.52
Type 1 or type 2 diabetes	7 (4)	2 (1)	0.09
Gestational diabetes	29 (14)	24 (10)	0.14
Marital status			<0.01
Married or cohabiting	153 (76)	218 (88)	
Single or widowed	13 (7)	30 (12)	
Unknown	35 (17)	1 (0.4)	

*Note:* Data are presented as means± standard deviations (SDs) or *N* (%).

^a^ *P* values correspond to *t*‐tests or Mann–Whitney *U* tests for continuous variables and *χ* ^2^ tests for categorical variables.

^b^ Data are presented as medians ± interquartile ranges.

Neither high nor low serum iodide was associated with stillbirth risk in unadjusted models or adjusted models (Table 2). For example, compared with serum iodide Quartiles 2 + 3 (*n* = 106 cases and 124 controls; range = 6.7–38.3 μg/L), the adjusted OR for Quartile 1 (*n* = 48 cases and 62 controls; range = 0.1–6.5 μg/L) was 0.92 (95% CI = 0.78–1.09) and for Quartile 4 (*n* = 45 cases and 63 controls; range = 38.3–228.3 μg/L) was 0.78 (95% CI = 0.45–1.33). Similarly, compared with normal Tg (≤75th percentile; *n* = 139 cases and 187 controls; range = 0.2–35.6 μg/L), high Tg (>75th percentile; *n* = 60 cases and 62 controls; range = 35.7–856.0 μg/L) was not strongly associated with risk of stillbirth in adjusted models (OR = 1.31 [95% CI = 0.82–2.10]). Neither high TSH (*n* = 8 cases and 8 controls; range = 3.4–29.7 mIU/L; OR = 0.84 [95% CI = 0.22–3.18]) nor low TSH (*n* = 6 cases and 12 controls; range = 0.0–0.2 mIU/L; OR = 0.84 [95% CI = 0.22–3.18]) was associated with risk of stillbirth, compared with normal TSH (*n* = 185 cases and 229 controls; range = 0.1–3.5 mIU/L). However, very few women had TSH values outside the population‐ and trimester‐specific normal reference range (*n* = 14 cases and 20 controls).

**Table 2:** Odds ratios (ORs) and 95% confidence intervals (CIs) for serum iodide, thyroglobulin and thyroid‐stimulating hormone and stillbirth: Finnish Maternity Cohort, 2012–2013

	*N* (cases:controls)	Median (min–max)	Unadjusted OR (95% CI)	Adjusted^a^ OR (95% CI)
Iodide				
Log(iodide)			0.98 (0.85–1.13)	0.92 (0.78–1.09)
Quartile (Q)1	48:62	3.4 (0.1–6.5)	0.91 (0.57–1.43)	1.14 (0.68–1.90)
Q2–Q3	106:124	18.8 (6.7–38.3)	1 (referent)	1 (referent)
Q4	45:63	57.9 (38.3–228.3)	0.84 (0.53–1.33)	0.78 (0.45–1.33)
Normal iodide (≥5th percentile)	188:237	21.3 (1.38–228.3)	1 (referent)	1 (referent)
Low iodide (<5th percentile)	11:12	0.84 (0.1–1.34)	1.14 (0.49–2.65)	1.26 (0.49–3.22)
Thyroglobulin (Tg)				
Log(Tg)			1.03 (0.87–1.23)	1.01 (0.83–1.24)
Normal Tg (≤75th percentile)	139:187	16.3 (0.2–35.6)	1 (referent)	1 (referent)
High Tg (>75th percentile)	60:62	53.4 (35.7–856.0)	1.30 (0.86–1.98)	1.31 (0.82–2.10)
Normal Tg (≤95th percentile)	187:237	20.4 (0.2–83.6)	1 (referent)	1 (referent)
Very high Tg (>95th percentile)	12:12	116.0 (85.1–856.0)	1.27 (0.56–2.89)	1.24 (0.49–3.11)
Thyroid‐stimulating hormone (TSH)^b^				
Log(TSH)			1.08 (0.89–1.30)	1.08 (0.86–1.36)
Low	6:12	0.04 (0.0–0.2)	0.68 (0.25–1.87)	0.47 (0.13–1.69)
Normal	185:229	1.1 (0.1–3.5)	1 (referent)	1 (referent)
High	8:8	4.1 (3.4–29.7)	1.25 (0.40–3.92)	0.84 (0.22–3.18)

^a^ Models adjusted for age, prepregnancy body mass index, socio‐economic status, smoking status, parity and marital status.

^b^ TSH levels were classified as low, normal and high based on gestational age at the time of sample collection. Low TSH is defined as <0.1 mIU/L in the first trimester and <0.2 mIU/L in the second trimester. Normal TSH is defined as 0.1–3.1 mIU/L in the first trimester and 0.2–3.5 mIU/L in the second trimester. High TSH is defined as >3.1 mIU/L in the first trimester and >3.5 mIU/L in the second trimester. Analyses excluded three cases and three controls with a known history of thyroid disease.

In sensitivity analyses excluding women with high or low TSH during their pregnancy, associations for serum iodide and stillbirth were substantively unchanged (data not shown). Further sensitivity analyses excluding women with pre‐eclampsia and gestational diabetes produced estimates similar to the overall analyses (data not shown). Restricted cubic spline models revealed no linear or non‐linear associations between serum iodide and stillbirth.

## 4 DISCUSSION

In this prospective study, we found that serum iodide, Tg and TSH, measured at 10–14 weeks gestation in Finnish pregnant women, were unrelated to risk of stillbirth. These findings are reassuring, given that (1) Finland halted its mandatory salt iodization programme in 1986 and intakes of iodine‐rich foods have steadily declined since; (2) the Finnish population is considered iodine insufficient and (3) pregnant women, generally, are at increased risk for iodine deficiency (Gizak et al., 2017; Ittermann et al., 2020; Leung et al., 2011; Nyström et al., 2016). Adequate iodine consumption during pregnancy remains important for prevention of conditions like congenital hypothyroidism (Pearce et al., 2016) and cretinism (Zimmermann et al., 2008), but mild to moderate iodine insufficiency does not appear to be importantly related to stillbirth risk among pregnant Finnish women, according to our results.

Previous studies on iodine and stillbirth have produced conflicting results (Charoenratana et al., 2016; Dillon & Milliez, 2000; Kemp, 1939; Oral et al., 2016; Potter et al., 1979; Yang et al., 2018). Our findings are in agreement with three studies reporting no association of iodine status and stillbirth (Charoenratana et al., 2016; Oral et al., 2016; Yang et al., 2018). For example, urinary iodine was not associated with history of stillbirth in a cross‐sectional study of 3543 women (Oral et al., 2016). Similarly, a prospective study of 2347 pregnancies among Chinese women reported no associations between UICs and risk of stillbirth (Yang et al., 2018), which is consistent with findings of another prospective study of 399 pregnant Thai women (Charoenratana et al., 2016). Notably, both prospective studies accrued fewer than 10 cases of stillbirth and therefore lacked the statistical power to draw any strong conclusions (Charoenratana et al., 2016; Yang et al., 2018).

In contrast, some other studies reported positive associations of iodine insufficiency or deficiency and risk of stillbirth (Dillon & Milliez, 2000; Kemp, 1939; Potter et al., 1979). For example, one study reported a decline in stillbirth rates as iodine supplementation programmes were introduced in Tasmania but did not account for the potential role of other aspects of nutrition, which were also likely contemporaneously improving (Potter et al., 1979). Likewise, Kemp qualitatively compared stillbirth rates in different Canadian populations and concluded that an iodine‐rich diet was associated with lower rates of stillbirth (Kemp, 1939). Another study reported an almost twofold risk of stillbirth among African women who were severely iodine deficient compared with nondeficient women (Dillon & Milliez, 2000). Importantly, 32% of the women in the study showed evidence of a goitre, indicating that hypothyroidism was common in the population. Discordant findings between these studies and ours may be explained by confounding bias arising from ecologic design (Kemp, 1939; Potter et al., 1979) and co‐occurring nutritional deficiencies (Dillon & Milliez, 2000). Differences in the prevalence of thyroid disease may also explain differences, as TSH levels were mostly normal and the prevalence of thyroid disease in our study population was low (0.4%).

Our study has several notable strengths. The population‐based nested case–control design of our study is considerably more efficient than prior prospective studies on this subject, given the rarity of stillbirth as a pregnancy outcome. Stillbirth cases and controls were randomly selected from among nearly all pregnant women in Finland during 2012–2013, and iodine status was assessed during 10–14 weeks gestation, limiting the potential for selection and information biases common to case–control studies. Finland is a developed country where mild to moderate iodine deficiency is more common than severe deficiency (Ittermann et al., 2020; Nyström et al., 2016; Viñas et al., 2011); thus, our study is an important addition to the literature because it may be more generalizable to pregnant women in developed countries, where milder iodine deficiency is common among pregnant women (Gizak et al., 2017). Furthermore, the nutritional status of the Finnish population is generally good, which reduces the likelihood that other nutritional deficiencies may confound our results. Additionally, we measured iodine levels in serum rather than urine. Urinary iodine has considerable intraindividual variation due to urine dilution, recent dietary intake and other factors (Als et al., 2000; Pan et al., 2019; Rasmussen et al., 1999). Urinary iodine is considered an acceptable biomarker of iodine status for whole populations but not for individuals (Konig et al., 2011). Serum iodide appears to be less sensitive to recent dietary intake and may thus better reflect individual iodine status over longer periods of time, reducing potential misclassification of exposure (Cui et al., 2019; Jin et al., 2017; Yu et al., 2018). Indeed, UIC and serum iodide have been shown to be strongly correlated; however, serum iodide has considerably higher accuracy than UIC for identifying individuals with thyroid dysfunction (Jin et al., 2017). In addition to serum iodide, we also examined levels of Tg and TSH, which are sensitive markers of functional iodine status (Stinca et al., 2017). Previous research suggests that median Tg < 13 μg/L and/or <3% of the population with Tg > 40 μg/L indicates iodine sufficiency among school‐aged children and adults (Ma et al., 2016; Zimmermann et al., 2013). Median Tg among healthy controls in our population (from whom we constructed the normal reference range) was 16.6 μg/L, suggesting that our study population is mildly to moderately iodine insufficient. The consistent lack of associations observed for serum iodide, in conjunction with those of Tg and TSH, collectively suggest that iodine status is unlikely to be an important risk factor for stillbirth, at least within the ranges observed among pregnant Finnish women.

Our study also has several limitations. First, we were only able to collect samples at one time point in pregnancy (at 10–14 weeks gestation). Changes in iodine status after sample collection due to changes in dietary intake or iodine supplementation may have contributed to intraindividual variability and misclassification of iodine status. Further, established reference ranges or cutpoints for serum iodide are not yet available; thus, we constructed two potential reference ranges for serum iodide in our population based on the distribution of serum iodide in healthy controls (Q2–Q3 range = 6.7–38.3 μg/L; ≥5th percentile: 1.34 μg/L). Finally, as in any observational study, our estimates are subject to potential residual confounding due to unmeasured factors, such as thyroid autoimmunity. Notably, very few women had TSH outside the normal range, suggesting that substantial confounding bias arising from thyroid autoimmunity is unlikely.

In conclusion, maternal iodine status in Finland, where mild to moderate iodine deficiency is prevalent (Ittermann et al., 2020; Nyström et al., 2016; Viñas et al., 2011), was not associated with risk for stillbirth in our study. TSH and Tg levels, which reflect functional iodine status, were also unrelated to stillbirth. Adequate iodine consumption during pregnancy is important to ensure adequate thyroid hormone production in the mother and fetus, but iodine status does not appear to affect risk of stillbirth. These findings provide reassurance that the degree of iodine insufficiency during pregnancy prevalent in developed countries is unlikely to increase risk of stillbirth.

## CONFLICTS OF INTEREST

The authors declare that they have no conflicts of interest.

## CONTRIBUTIONS

JLM and TM formulated the research question and designed the study. ES, H‐MS, MG, KK and U‐JK conducted the study. TM, ACP‐S and JLM analysed the data. ACP‐S, ER and JLM wrote the manuscript. All authors have read and approved the final manuscript.

## Data Availability

The data supporting the findings of this study have been obtained from the Finnish National Institute of Health and Welfare, which is the holder of the Medical Birth Register, and used under licence for the current study and so are not publicly available. The data cannot be made available to ensure privacy protection of the cohort members.
